# Cellulose Acetate and Supercritical Carbon Dioxide: Membranes, Nanoparticles, Microparticles and Nanostructured Filaments

**DOI:** 10.3390/polym12010162

**Published:** 2020-01-08

**Authors:** Stefano Cardea, Iolanda De Marco

**Affiliations:** Department of Industrial Engineering, University of Salerno, Via Giovanni Paolo II, 132, 84084 Fisciano (SA), Italy; scardea@unisa.it

**Keywords:** supercritical antisolvent process, supercritical CO_2_ phase inversion process, microporous membranes, micro and nanoparticles, nanostructured filaments

## Abstract

Cellulose acetate (CA) is a very versatile biocompatible polymer used in various industrial sectors. Therefore, depending on the application, different morphologies are required. Different processes at industrial scale are commonly employed to obtain CA micro or nanoparticles (discontinuous structures) or CA membranes (continuous structures with discontinuities). In this work, two supercritical carbon dioxide (scCO_2_) based techniques, such as the semi-continuous supercritical antisolvent process (SAS) and the supercritical fluid phase inversion process, in which scCO_2_ plays the role of antisolvent, were employed. Varying the kind of organic solvent used to prepare the polymeric solution, the polymer concentration, and operating pressure and temperature, it was possible to tune the characteristics of the obtained material. In particular, using acetone as the organic solvent, filaments constituted by nanoparticles, expanded microparticles, nanoparticles with a mean diameter lower than 80 nm, and microporous membranes were obtained, varying the operating conditions. The attainment of spherical micron-sized particles was instead achieved using a mixture of acetone and DMSO as the organic solvent. Therefore, the versatility of the supercritical carbon dioxide-based techniques has been confirmed, and it was possible to obtain, using a single experimental plant, various morphologies of cellulose acetate (with controllable particles’ or pores’ diameters) by varying the operating conditions.

## 1. Introduction

Different techniques have been developed to obtain polymeric materials in form of micro and nanoparticles, fibers, foams, scaffolds and membranes to be used in several industrial applications (food industry, tissue engineering, mechanical engineering, biomedical field, pharmaceuticals, chemical engineering, etc.). Among them, spray drying [1], electrospinning [2], chemical foaming [3], gel drying [4], and phase inversion [5] are some of the most studied and diffused processes, but they are also characterized by some crucial limitations. For example:

- the spray drying process is characterized by variations in particle shape and particle size distribution, high process temperatures, and high drying speeds that normally do not allow the encapsulation of temperature-sensitive bioactive substances [6];
- the electrospinning process is characterized by the use of organic solvents that can be toxic, difficulty in obtaining 3-D structures as well as sufficient size of pores needed for biomedical applications and suitable mechanical behavior; moreover, the process depends on a high number of variables and their combination (humidity, temperatures, collector distance, high voltage, etc.) [7];
- the chemical foaming process is simple, but high mold manufacturing precision is required (the mold cost is high), and a second clamping pressure device is needed during high-pressure foaming process [8];
- the gel drying process is characterized by a high cost of the raw materials (the chemicals) and there is often a large volume shrinkage and cracking during the drying step (the removing of the “organics” can cause the collapse of the structure due to their surface tension); moreover, long processing times can be necessary [9];
- the phase inversion process is characterized by the use of organic solvents and necessity of post-treatments on the generated material, low versatility (once the system polymer-solvent-nonsolvent is selected, only one kind of morphology is possible), long processing times (from several hours to days), etc. [5].

For these reasons, in the last thirty years, the supercritical fluids have been tested to overcome some of the limitations reported above, obtaining successful results [10,11,12]. Indeed, supercritical fluids have some peculiar characteristics, such as liquid-like densities and gas-like diffusivities, which allow to largely improve the performances of the above-mentioned processes. Furthermore, low residual solvents are present in the final structures due to the high affinity between scCO_2_ and most of the organic solvents normally used in the processes. Then, it is possible to recover the solvents at the end of the processes, simply depressurizing the plant, thus minimizing the pollution due to the processes.

The most used substance at supercritical conditions is carbon dioxide (scCO_2_), for its readily accessible critical pressure and temperature, its non-toxicity, non-flammability, and for its cheapness [13,14,15,16].

The scCO_2_ based processes applied to polymeric materials may be classified into two main subsets:

- attainment of a discontinuous structure (micro and nanoparticles, for example);
- attainment of a continuous structure with discontinuities (such as fibers, membranes, foams, and scaffolds).

In the case of a discontinuous structure, different processes have been developed which can be classified according to the different roles played by scCO_2_. In particular, scCO_2_ can solubilize the polymer (RESS, rapid expansion of supercritical solution) [17], it can play the role of antisolvent (SAS, supercritical antisolvent [18] and GAS, gas antisolvent [19]), the role of co-solute (SAA, supercritical assisted atomization [20] and PGSS, particles from gas-saturated solutions [21]) or it can extract the oily phase from oil-in-water emulsions [22]. Among them, one of the most used is the SAS process that, compared to the traditional micro and nanoparticles formation processes (i.e., spray drying, etc.), allows to obtain better results in terms of particle shape and size distribution, using low processing temperatures (i.e., 35–45 °C). Moreover, thanks to the scCO_2_ peculiarities, a high encapsulation rate of active substances in polymeric particles can also be achieved.

When the structure is continuous and shows some discontinuities, SAS process has been used to produce fibers [23], supercritical CO_2_ assisted phase inversion method has been used to obtain membranes [24], supercritical foaming to produce foams [25], and supercritical gel drying to produce scaffolds [26]. With respect to the traditional processes for the generation of continuous structures (gel drying, phase inversion, foaming, etc.), the scCO_2_ assisted processes allow to obtain better results in terms of versatility. Indeed, it is possible to control the final morphology of the structures simply varying the process parameters, such as temperature, pressure and processing times. Finally, the absence of surface tension at supercritical state allows to eliminate the solvents without collapsing the porous structures.

In this work, two scCO_2_ based techniques will be used (SAS and scCO_2_ assisted phase inversion). The two techniques have many points in common and a significant difference:

1. in both of them, the carbon dioxide acts as an antisolvent and flows continuously into the vessel;
2. the techniques are both in a batch mode with respect to the solid phase (a depressurization is necessary to recover the solute after the process);
3. the SAS process is continuous with respect to the polymeric solution that is injected through a nozzle in the supercritical medium, whereas, the scCO_2_ assisted phase inversion is batch, considering that the polymeric solution is charged at atmospheric pressure at the beginning of the experiment.

Cellulose acetate (CA), the acetate ester of cellulose, is a biocompatible polymer widely used for many industrial products, like textiles, filters, foils, photographic and motion pictures, films, moldings, coatings, membranes, LCD-displays, and controlled drug delivery systems [27,28,29,30]. This polymer is often used in the preparation of semipermeable membranes for dialysis, ultrafiltration, and reverse osmosis [28,31]. CA membranes have very low absorption characteristics (high throughput) and thermal stability with high flow rates. CA is also used as the carrier for the delivery of vitamins or pharmaceutical products both in the form of nanofibers [23,32,33,34] and microparticles [35,36]. Moreover, CA nanoparticles are used in novel applications to modify bulk materials, because, if compared to bulk material, cellulose acetate nanoparticles offer a huge surface area which is useful to trigger selectively surface interactions, and hence design materials with novel properties [37].

Considering the multiplicity of morphologies for which cellulose acetate can be used for different applications, their attainment using a single technique or similar techniques that share a single plant would be interesting. Indeed, it is clear from the literature analysis that the different CA morphologies were obtained using very disparate techniques ranging from electrospinning to nanoprecipitation to phase inversion [32,33,34,35,36,37].

Therefore, the purpose of this work is to obtain, using the aforementioned supercritical carbon dioxide-based techniques, different CA morphologies, simply by changing the operating conditions, such as pressure, temperature, and concentration of the polymeric solution. The SAS process will be applied in correspondence of concentrations of the polymer in the organic solvent which allows the jet break-up of the liquid solution. At high concentrations of the polymeric solution, in correspondence of which high viscosities prevent the liquid jet break-up, the scCO_2_ assisted phase inversion will be used.

Cellulose acetate is freely soluble in acetone, which is classified as a low-risk solvent to human health (class 3) [38] and, therefore, it will be the preferred solvent for the majority of the experiments. For some SAS experiments, a mixture of acetone and dimethylsulfoxide (DMSO) will be used as the organic solvent, because the jet fluid dynamics of acetone and DMSO is very different.

## 2. Apparatus, Materials and Methods

### 2.1. Materials

Cellulose acetate (CA, degree of substitution = 2.5, average M_n_ ≈ 50,000), dimethylsulfoxide (DMSO, purity 99.5%) and acetone (AC, purity 99.8%) were supplied by Sigma–Aldrich (Milan, Italy). Carbon dioxide (purity 99%) was purchased from Morlando Group (Sant’Antimo—NA, Italy). All materials were used as received. CA solubilities in the organic solvents were measured by stirring the solutions at 40 °C; they are equal to 25 mg/mL in DMSO and 320 mg/mL in AC.

### 2.2. Apparatus and Procedures

#### 2.2.1. Apparatus

The bench-scale plant used for all the experiments is a home-made apparatus equipped with a 316 stainless steel cylindrical high-pressure vessel with an internal volume of 500 mL (I.D. 5 cm). In the case of the SAS semi-continuous process, two pumps are used to deliver the liquid solution and the carbon dioxide, whereas, in the case of membrane preparation, only the pump for the carbon dioxide is used. scCO_2_ is pumped through an inlet port located on the top of the chamber. CO_2_ is heated to the process temperature before entering the precipitator. The precipitator is electrically heated using thin band heaters. The pressure in the chamber is measured using a test gauge manometer and regulated by a micrometric valve located at the exit (bottom) of the chamber. In the case of the SAS process, the liquid mixture is sprayed in the precipitator through a 200 μm thin wall stainless steel nozzle. A second vessel located downstream of the micrometric valve is used to recover the liquid solvent. At the exit of the second vessel, a rotameter and a dry test meter are used to measure the CO_2_ flow rate and the total quantity of antisolvent delivered, respectively.

#### 2.2.2. Procedure for SAS Experiments

The SAS experiments were performed at pressures ranging between 9 and 15 MPa, concentrations between 10 and 30 mg/mL, and temperatures in the range 35–60 °C. A SAS experiment started by delivering carbon dioxide at a constant flow rate to the precipitation chamber until the desired pressure was reached. The antisolvent steady flow was established, opening the micrometric valve. Then, the pure solvent was sent through the injector to the pressurized chamber, with the aim of obtaining steady-state composition conditions during the solute precipitation. At this point, the flow of the organic solvent was stopped and the liquid solution, formed by the organic solvent and the polymer, was delivered through the nozzle, at the same flow rate of the pure solvent. At the end of the precipitation process, scCO_2_ continued to flow to wash the chamber from the residual content of liquid solubilized in the supercritical antisolvent. A stainless-steel frit is put at the bottom of the chamber to collect the solid product, allowing the CO_2_–organic solvent solution to pass through. At the end of the washing step, CO_2_ flow was stopped, and the precipitator was slowly depressurized down to atmospheric pressure.

#### 2.2.3. Procedure for Membranes’ Preparation

Homogenous solutions of cellulose acetate in acetone were prepared in the range of polymer concentrations between 40 and 320 mg/mL by stirring at 40 °C. The solution was placed in a formation cell (steel caps with a diameter of 2.5 cm and heights ranging from 300 to 600 μm) spreading it with a glass stick to control the thickness of the film. The cell was rapidly put inside the vessel to avoid the evaporation of acetone. The vessel was then filled with scCO_2_ up to the desired pressure. In the first part of the process, CO_2_ penetrates in the solution operating in batch mode for 1 h. After this period of time, a micrometric valve is opened and we operated in continuous mode; i.e., with a constant CO_2_ flow rate set at 1.2 kg/h, holding pressure and temperature constant to dry the phase-separated membrane for 2 h. Then, the vessel was slowly depressurized for 2 h.

### 2.3. Characterization

Samples of the precipitated powder were collected at different points inside the precipitation chamber and examined using a Field Emission Scanning Electron Microscope (FESEM, mod. LEO 1525, Carl Zeiss SMT AG, Oberkochen, Germany). FESEM samples were covered with 250 A of gold using a sputter coater (mod. 108A, Agar Scientific, Stansted, UK).

Particle size (PS) and particle size distribution (PSD) were measured using an image processing software (SigmaScan Pro, Jandel Scientific, Bangalore, India) that counts, measures, and analyzes digital images.

Thermograms of CA were obtained using a Differential Scanning Calorimeter (DSC, mod. TC11, Mettler-Toledo, Inc., Columbus, OH, USA) using Mettler STARe system. Fusion temperature and enthalpy were previously calibrated with indium standard materials (melting point 156.6 °C, enthalpy of fusion 28.52 J/g). CA powder samples (5 ± 0.5 mg), prepared in duplicates, were accurately weighed, crimped into an aluminum pan and heated from 25 to 250 °C at 10 °C/min under a nitrogen purge (50 mL/min).

## 3. Results and Discussion

### 3.1. Supercritical Antisolvent Precipitation

The operating conditions of the experimental plan were chosen on the basis of our expertise in the SAS process. In particular, the pressure was varied between 9 and 15 MPa and the temperature between 35 and 60 °C. As regards the choice of pressure, it is well-known that at pressures in the proximity of the mixture critical point (MCP) of the solvent-carbon dioxide system, microparticles are generally obtained, while, at pressures quite higher than MCP, nanoparticles precipitate. Therefore, the minimum pressure value was set at 9 MPa. This value is about 1.5 MPa above the MCP of the binary system to be sure to locate the operating point above the MCP of the ternary system formed by the polymer, the solvent and the antisolvent; i.e., to take into account that the addition of the polymer can shift the MCP towards higher pressures [39,40]. The upper end of the range (15 MPa) is well above the MCP; in correspondence of this region, fully developed supercritical conditions are realized, the surface tension of the liquid in contact with the scCO_2_ disappears before the liquid jet break-up in droplets and the solute precipitation occurs from a gas plume. As regards the temperature, the minimum value (35 °C) was chosen just above the carbon dioxide critical temperature, which is equal to 31.1 °C, while the maximum one was fixed at 60 °C. Higher temperatures would cause an unnecessary increase in process costs, without a gain in terms of performance of the process itself. The liquid solution flow rate was fixed at 1 mL/min, the ratio between CO_2_ flow rate and liquid flow rate (R) was set equal to 30 on a mass basis and the CO_2_ molar fraction was equal to 0.98.

A list of all the performed experiments with the operating conditions and the obtained morphologies is reported in Table 1. The mean diameter (m.d.) and the standard deviation (s.d.) are related to the nanoelements constituting the filaments in the case of nanostructured filaments (NF), and to the particles in the case of nanoparticles (NP), microparticles (MP), and expanded microparticles (EMP).

#### 3.1.1. AC as the Liquid Solvent

**Figure 1:**
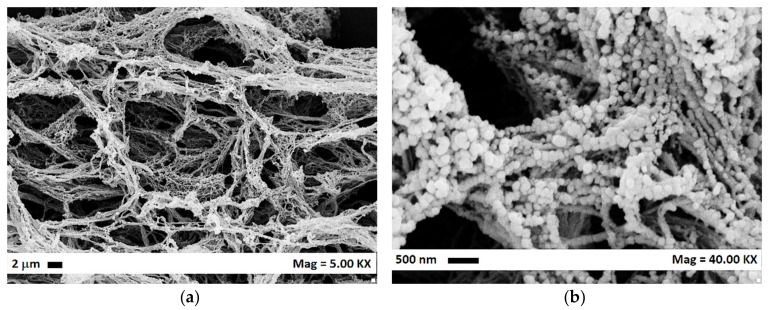
CA filaments obtained at 12 MPa, 35 °C and 10 mg/mL; (**a**) FESEM image at 5 KX; (**b**) FESEM image at 40 KX.

Using AC as the liquid solvent, the effect of the operating pressure and temperature on the morphology of the precipitated polymer was studied at different concentrations. The first set of experiments (#1–7) was performed at 10 mg/mL, varying the temperature from 35 to 50 °C and the pressure from 9 to 15 MPa. We observed that, at 9 and 12 MPa, for all the temperatures, nanostructured filaments were recovered in correspondence of the nozzle. It is possible to observe from Figure 1a,b, in which exemplificative FESEM images were reported, that the filaments are constituted by nanometric elements. It is possible to deduce that the viscous forces are predominant, and the jet break-up did not occur.

Increasing the pressure at 15 MPa (#5–7), in correspondence of all the tested temperatures, nanoparticles with mean diameters lower than 80 nm were precipitated. From the values of mean particle diameters and standard deviations reported in Table 1, it is possible to observe that increasing the temperature a slight increase in the mean diameter occurred.

**Figure 2:**
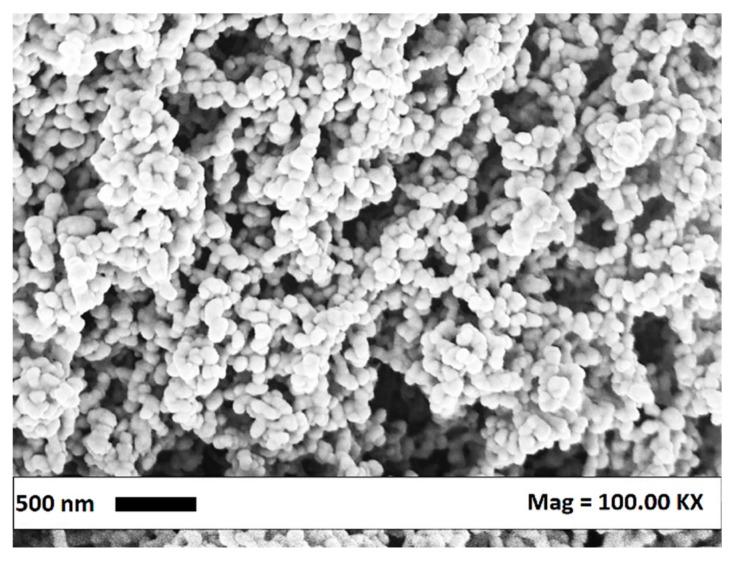
CA nanoparticles obtained at 15 MPa, 60 °C and 15 mg/mL.

The subsequent set of experiments (#8–15) was performed fixing the CA concentration in AC at 15 mg/mL. The observed trend is analogous to the one obtained at 10 mg/mL. At 12 MPa, nanostructured filaments were obtained, whereas at 15 MPa nanoparticles were obtained (see Figure 2 for an exemplificative FESEM image). In correspondence of the lower pressure and the higher temperature (9 MPa and 60 °C), the polymer precipitated in form of expanded microparticles with a mean diameter equal to about 16 µm. This morphology can be ascribed to the solute precipitation from a subcritical phase at high carbon dioxide molar fractions. In this case, the operating point is, in a pressure vs carbon dioxide molar fraction diagram, located on the right of the miscibility hole at pressures lower than the MCP pressure. Indeed, as stated in the first part of the Section 3.1, the presence of the polymer can shift the MCP towards higher pressures. This means that the operating point at 9 MPa can lie in the subcritical region.

**Figure 3:**
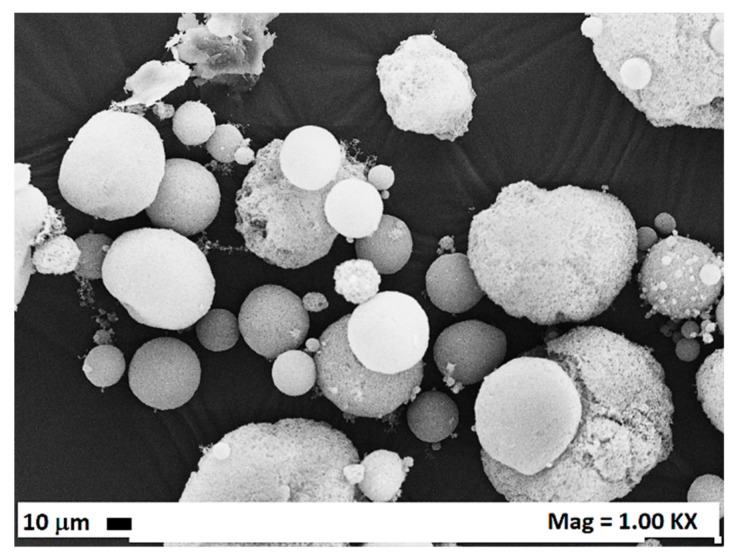
Expanded microparticles obtained at 9 MPa, 60 °C and 20 mg/mL.

A further increase of concentration at 20 and 30 mg/mL (#16–21) confirmed the results obtained at lower concentrations: EMP were precipitated at 9 MPa and nanostructured filaments at higher pressures. An exemplificative FESEM image of the EMP constituted by nanometric elements is reported in Figure 3. It is possible to observe that nanoparticles were not precipitated at concentrations higher than 20 mg/mL, even fixing the pressure at 15 MPa. This is ascribable to the high quantity of polymer present in the solution. Indeed, the particles are obtained because, when the liquid at the exit of the nozzle enters in contact with the scCO_2_, the surface tension is quickly reduced to zero: droplets are not formed because the time required for the zeroing of the surface tension is less than the jet break-up time and nanoparticles precipitated from the gas plume through nucleation and growth mechanisms [41]. At higher concentrations (20 and 30 mg/mL), which means higher viscosities of the liquid solution, the precipitated nanoparticles are forced by the cohesive effect of the viscosity to form nanostructured filaments.

#### 3.1.2. AC/DMSO as the Liquid Solvent

**Figure 4:**
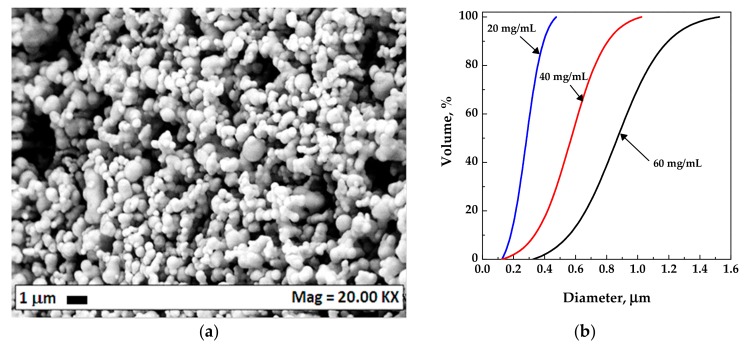
Microparticles obtained at 9 MPa, 40 °C; (**a**) exemplificative FESEM image for the particles precipitated at 40 mg/mL; (**b**) particle size distributions with the effect of the concentration.

Considering that it is well known from the literature that microparticles are difficultly obtained using AC as the organic solvent, because of the very sharp transition from two-phases to one-phase behavior [42], CA was precipitated from mixtures constituted by AC/DMSO 50/50 *v*/*v*, with the aim of obtaining microparticles. Indeed, differently from AC, DMSO is characterized by a wide transition from two-phases to one-phase behavior and is one of the preferred solvents for the attainment of microparticles using the SAS process. For this set of experiments (#22–24), the operating pressure and temperature were fixed at 9 MPa and 40 °C respectively and the effect of CA concentration in the liquid solvent mixture was investigated. As it is possible to observe from the mean diameters and from the standard deviation values reported in Table 1, by increasing the concentration, the mean particle size increased, and the particle size distribution enlarged. In Figure 4, an exemplificative FESEM image (microparticles obtained at 40 mg/mL) and the volumetric cumulative particle size distributions of the microparticles obtained from the experiments #22–24 are reported.

### 3.2. Supercritical Phase Inversion Experiments

At concentrations higher than 30 mg/mL, it was not possible to inject the liquid solution inside the pressurized vessel because the nozzle was blocked from the too high viscosity of the solution. Therefore, at polymer concentrations from 40 to 320 mg/mL, supercritical phase inversion experiments were performed fixing the temperature at 45 °C and varying the pressure from 8 to 20 MPa, as reported in Table 2. The experimental parameters were chosen in agreement on the results obtained using similar polymeric solutions [24]. In particular, polymer concentration is usually comprised between 30 and 350 mg/mL, processing temperature between 35 and 55 °C and processing pressure between 8 and 25 MPa. Analyzing previous results on the supercritical phase inversion process [24,30], we verified as polymer concentration is a crucial parameter of the process, whereas the increase of temperature and the decrease of pressure leads to similar trends in terms of membranes morphology modification. For this reason, in this work, a large range of polymer concentration was explored (i.e., from 40 to 320 mg/mL) and only one parameter among temperature and pressure was varied during the experiments: temperature was fixed at 45 °C, that is a good compromise between scCO_2_ solvent power and diffusivity, whereas processing pressure was varied between 8 and 20 MPa, that is a standard operative range of the supercritical phase inversion process. As expected, changing the process parameters, we verified that it was possible to obtain different membranes morphologies. Indeed, as reported in previous works [24,30], polymer concentration, pressure, and temperature can affect the phase inversion mechanisms.

**Figure 5:**
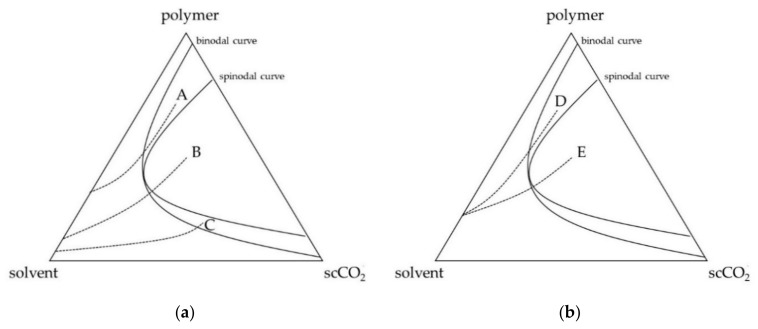
Qualitative ternary diagrams; (**a**) paths at different starting polymer concentrations at 8 MPa and 45 °C; (**b**) paths at different operating pressures at 160 mg/mL and 45 °C.

In particular, to understand the effect of the process parameters on the membranes’ morphologies, it is important to consider the ternary diagram formed by polymer, solvent and scCO_2_ and its miscibility hole, formed by an external binodal curve and an internal spinodal curve, as qualitatively represented in Figure 5a,b.

**Figure 6:**
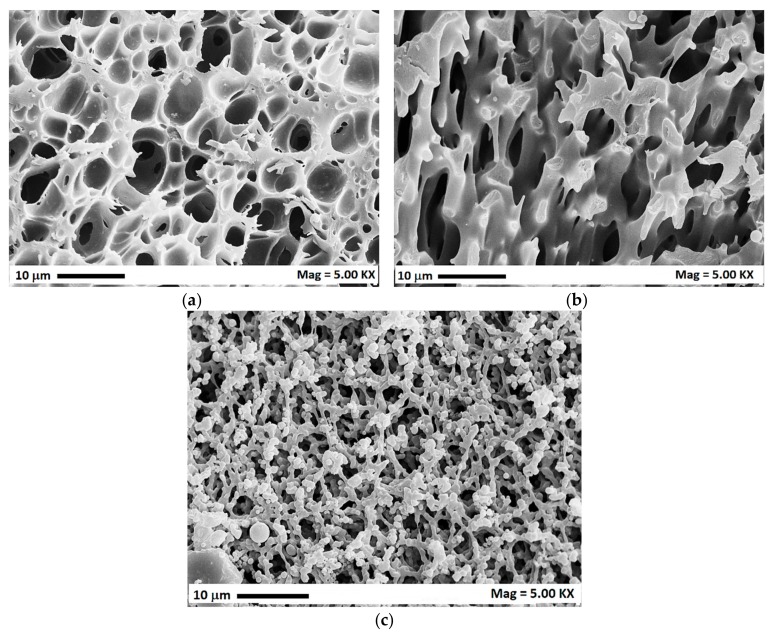
Different membrane morphologies obtained at 8 MPa, 45 °C and different polymer concentrations (40, 80 e 240 mg/mL): (**a**) cellular membrane; (**b**) spinodal membrane; (**c**) beads-like membrane.

Indeed, depending on the demixing point, i.e., the point inside the miscibility hole in which the phase separation occurs, different morphologies can be obtained. The location of this point is related to process parameters:

(a) starting from a high polymer concentration solution, the demixing point will be located in the upper part of the miscibility hole between the binodal and spinodal curves (point A in Figure 5a) and a liquid-liquid binodal demixing with nucleation and growth of the polymer-lean phase inside the polymer-rich phase occurs, generating a cellular structure membrane (CM). An examplificative FESEM image is reported in Figure 6a;
(b) decreasing the polymer concentration of the starting solution, the phase inversion occurs in the central part of the demixing hole of the ternary diagram in which the demixing point is located inside the spinodal curve (point B in Figure 5a), where a spinodal demixing is favored, leading to the generation of a spinodal membrane morphology (SM). An example of this morphology is reported in Figure 6b;
(c) starting from low polymer concentration solutions, the demixing point is located in the lower part of the miscibility hole, again between the binodal and spinodal curves (point C in Figure 5a); a liquid-liquid binodal demixing with nucleation and growth of the polymer-rich phase inside the polymer-lean phase is favored, leading to the formation of a bead-like membrane (BLM). An examplificative FESEM image is reported in Figure 6c.

It is possible to observe that also the variation of pressure (and temperature) can affect the final membranes’ morphology. Indeed, the pressure and temperature can act both on the miscibility hole area and shape inside the ternary diagram polymer-solvent-scCO_2_, and on the kinetics of the phase inversion; i.e., an increase of scCO_2_ solvent power (at higher pressure and lower temperature) can cause a modification of the ternary solution pathway inside the ternary diagram, leading to the formation of different morphologies. From Table 2, it is possible to observe that, starting from the same polymer concentration (for example 160 mg/mL), different morphologies can be obtained (CM and SM), simply varying the operating pressure from 10 to 20 MPa; a qualitative representation of this result is reported in Figure 5b. It is possible to suppose that, starting from the same polymer concentration, an increase of the pressure leads to a modification of the trajectory of the concentration pathway, and a consequent shift of the demixing point: operating at 10 MPa, the process is slower and the trajectory goes towards the polymer apex leading to the formation of a cellular structure (point D in Figure 5b); on the other hand, working at 20 MPa, the demixing process becomes faster and the trajectory tends to largely go towards the scCO_2_ apex, leading to the formation of a spinodal membrane (point E in Figure 5b). A further increase of the operating pressure could theoretically lead to the formation of microparticles, as already verified in previous works [24,30]. Then, in this experimentation, it is possible to suppose that the pressure affects the kinetics of the process, modifying the concentration pathways inside the ternary diagram of the system.

These results confirmed the high versatility of the scCO_2_ assisted phase inversion process, differently from what occurs with the traditional phase inversion methods.

## 4. Analyses

**Figure 7:**
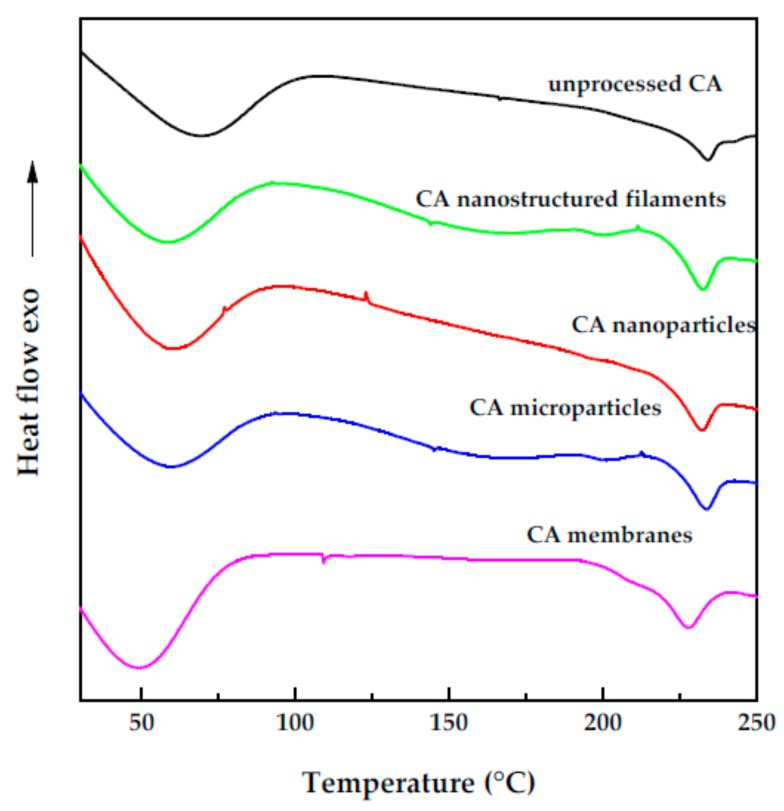
DSC thermograms for unprocessed CA, SAS processed CA and CA membranes; exo indicates exothermic flow.

DSC thermograms of untreated cellulose acetate, SAS processed CA microparticles, nanoparticles and filaments, and phase inversion membranes are shown in Figure 7. A broad endothermic event between the ambient temperature and 100 °C is evident in all the DSC traces and is ascribable to the water desorption from the polymer. A second endothermic peak at about 230 °C can be visualized for all the traces and is related to the melting of the polymer. The DSC analysis indicated that neither the SAS process nor the phase inversion method altered the product structure.

## 5. Conclusions

Supercritical carbon dioxide-based techniques were applied to obtain different morphologies of cellulose acetate, using acetone or a mixture of acetone and DMSO as the liquid solvent. The versatility of the supercritical antisolvent based processes was confirmed. Indeed, nanoparticles, microparticles, nanostructured filaments, and membranes with different morphologies and pore structures were obtained with a unique plant simply varying the operating pressure, temperature, and concentration of the liquid solution.

## Figures and Tables

**Table 1:** List of the performed experiments using supercritical antisolvent precipitation. NF: nanostructured filaments; NP: nanoparticles; MP: microparticles; EMP: expanded microparticles.

#	c, mg/mL	P, MPa	T, °C	Morph.	m.d. ± s.d., nm	Figure
AC as the liquid solvent						
1	10	9	40	NF	92 ± 22	
2		12	35	NF	78 ± 18	Figure 1a,b
3			40	NF	80 ± 20	
4			50	NF	108 ± 21	
5		15	35	NP	76 ± 14	
6			40	NP	78 ± 15	
7			50	NP	82 ± 19	
8	15	9	60	EMP	16,070 ± 6170	
9		12	35	NF	100 ± 22	
10			40	NF	108 ± 24	
11			50	NF	110 ± 24	
12			60	NF	114 ± 26	
13		15	35	NP	78 ± 14	
14			50	NP	80 ± 15	
15			60	NP	83 ± 16	Figure 2
16	20	9	60	EMP	30,600 ± 15,230	Figure 3
17		12	40	NF	109 ± 25	
18		15	40	NF	102 ± 22	
19	30	9	60	EMP	31,300 ± 16,200	
20		12	40	NF	132 ± 28	
21		15	40	NF	112 ± 23	
AC/DMSO 50/50 as the liquid solvent						
22	20	9	40	MP	232 ± 50	
23	40			MP	403 ± 160	Figure 4
24	60			MP	670 ± 60	

**Table 2:** List of the performed experiments using supercritical phase inversion. CM: cellular membrane; SM: spinodal membrane; BLM: beads-like membrane.

#	c, mg/mL	P, MPa	T, °C	Morph.	Figure
25	40	8	45	BLM	Figure 6c
26		10		BLM	
27		20		BLM	
28	80	8	45	SM	Figure 6b
29		10		SM	
30		20		SM	
31	160	8	45	CM	
32		10		CM	
33		20		SM	
34	240	8	45	CM	Figure 6a
35		10		CM	
36	320	10	45	CM	
